# Knowledge brokers and how to communicate knowledge in 2010

**DOI:** 10.1186/1710-1492-6-S4-A3

**Published:** 2010-12-10

**Authors:** Saliha Ziam

**Affiliations:** 1 Teluq-University of Québec in Montréal, Québec (Head Office), G1K 9H6, Canada

## 

The importance of using healthcare evidence by policy-makers is widely recognized [1,2]. For over a decade, several strategies to improve the use of knowledge by policy makers have been promoted [3,4]. Among them, the use of individuals called “intermediaries” or “knowledge brokers” is presented as a potential strategy [5,6]. Situated at the organizational interface, these actors benefit from a strategic position allowing easier access to external knowledge [7]. Therefore, they must develop sufficient skills to be able to properly take profit of all opportunities to create the value for their organization. In fact, many authors consider brokers as true knowledge integrators that assess, interpret, synthesize, exploit and transfer relevant knowledge. Despite the availability of several studies that stress the importance of the multifaceted role of brokers, few have explored how they concretely integrate or “absorb” knowledge and especially, which skills are necessary to ensure the success of such activities.

We propose a new conceptual model on research integration by knowledge brokers and provide an empirical testing of this proposed model. This conceptual framework (figure 1) builds upon recent theoretical developments on the concept of knowledge absorptive capacity [8]*i.e.,* starting from the following dimensions: knowledge identification (recognize value of new knowledge), acquisition, assimilation, transformation and knowledge exploitation. To test the conceptual framework, we collected survey data. The sample of 297 respondents included members of the knowledge brokerage community of practice (CoP). Data analysis allowed presenting a first portrait of the profile of knowledge brokers working in health organizations in Canada. In this perspective, several descriptive analyses, such as the distribution of knowledge brokers according to their membership organizations, their status, education (last Diploma), experience, *etc.*, were completed. The bivariate analyses used these dimensions to compare knowledge brokers regarding their knowledge absorptive capacity and the explanatory variables documented in the literature. These results show that the brokers’ absorptive capacity of knowledge improved with higher levels of advanced education attainment, such as a PhD. However, employment status of brokers and their skills was also a factor affecting knowledge broker performance. It was found that brokers with professional status identify more knowledge than do brokers with senior managers’ status. Conversely, brokers with senior manager status transform and exploit knowledge more effectively than do brokers with professional status. Also, other organizational factors that act as facilitators or barriers to knowledge absorption by brokers are the organizational unit size where they perform their daily tasks. We found that the brokers who are assigned to medium size units are more able to identify, assimilate, transform and create opportunities of knowledge exchange in their workplace (*i.e.,* actively exploit knowledge). Finally, these results allow us to establish the differences between the brokers according to their level of advanced education, status, organizational affiliation, and the size of the organizational unit. Further multivariate analyses are needed to identify all the factors associated with brokers’ absorptive capacity.

**Figure 1:**
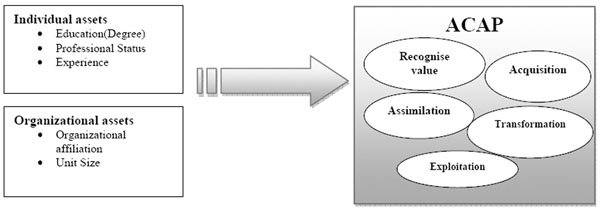
Theoretical framework of the absorptive capacity of knowledge brokers adapted from Todorova & Durisin (2007)

## References

[B1] Lavis JN Research, public policymaking, and knowledge-translation processes: Canadian efforts to build bridges J Contin Educ Health Prof 2006 26 37 45 10.1002/chp.49 16557509

[B2] Ward V A House Developing a framework for transferring knowledge into action: a thematic analysis of the literature J Health Services Research & Policy 2009 14 156 164 10.1258/jhsrp.2009.008120 PMC2933505 19541874

[B3] Landry R Amara N Pablos-Mendes A Shademani R Gold I The knowledge-value chain: A conceptual framework for knowledge translation in health Bull World Health Organ 2006 84 597 602 16917645 10.2471/BLT.06.031724 PMC2627427

[B4] Amara N Ouimet M Landry R New Evidence on Instrumental, Conceptual and Symbolic Utilization of University Research in Government Agencies Science Communication 2004 26 75 106 10.1177/1075547004267491

[B5] Hargadon A Brokering knowledge: Linking learning and innovation Research in Organizational behaviour 2002 24 41 85 10.1016/S0191-3085(02)24003-4

[B6] Lomas J The in-between world of knowledge brokering BMJ 2007 334 129 132 17235094 10.1136/bmj.39038.593380.AE PMC1779881

[B7] Cohen WM Levinthal DA Absorptive Capacity: A New Perspective on Learning and Innovation Administrative Science Quarterly 1990 35 1 128 152 10.2307/2393553

[B8] Todorova G Durisin B Absorptive Capacity: Valuing a Reconceptualization The Academy of Management Review 2007 32 774

